# Silica and Selenium Nanoparticles Attract or Repel Scale Insects by Altering Physicochemical Leaf Traits

**DOI:** 10.3390/plants13070952

**Published:** 2024-03-25

**Authors:** Siyi Gao, Midori Tuda

**Affiliations:** 1 Laboratory of Insect Natural Enemies, Graduate School of Bioresource and Bioenvironmental Sciences, Kyushu University, Fukuoka 8190395, Japan; 2 Laboratory of Insect Natural Enemies, Institute of Biological Control, Faculty of Agriculture, Kyushu University, Fukuoka 8190395, Japan

**Keywords:** Rutaceae, Diaspididae, silicon dioxide, nanotechnology, IPM, fruit tree, *Unaspis yanonensis*, *Citrus unshiu*, nanofertilizer, nanopesticide

## Abstract

Although nanoparticles have gained attention as efficient alternatives to conventional agricultural chemicals, there is limited knowledge regarding their effects on herbivorous insect behavior and plant physicochemistry. Here, we investigated the effects of foliar applications of nano-silica (SiO_2_NPs) and nano-selenium (SeNPs), and bulk-size silica (SiO_2_) on the choice behavior of the arrowhead scale insect on mandarin orange plants. One leaf of a bifoliate pair was treated with one of the three chemicals, while the other was treated with water (control). The respective SiO_2_, SeO_2_, calcium (Ca), and carbon (C) content levels in the leaf epidermis and mesophyll were quantified using SEM–EDX (or SEM–EDS); leaf toughness and the arrowhead scale density and body size were measured. First-instar nymphs preferred silica-treated leaves and avoided SeNP-treated leaves. SiO_2_ content did not differ between control and SiO_2_NP-treated leaves, but was higher in bulk-size SiO_2_-treated leaves. The SiO_2_ level in the control leaves was higher in the SiO_2_NP treatment compared with that in the control leaves in the bulk-size SiO_2_ treatment. Silica-treated leaves increased in toughness, but SeNP-treated leaves did not; leaf toughness increased with mesophyllic SiO_2_ content. The insect density per leaf increased with leaf toughness, SiO_2_ content and, in the SiO_2_NP treatment, with epidermal C content. There was no correlation between SeO_2_ content and insect density. This study highlights the potential uses of SeNPs as an insect deterrent and of silica for enhancing leaf toughness and attracting scale insects.

## 1. Introduction

Citrus, recognized globally as a key fruit crop, offers various health benefits due to its richness in nutrients that reduce the risk of cardiovascular and liver deficiencies and cancers [1,2]. Efficient and sustainable agriculture, characterized by a reduced reliance on chemical fertilizers, has prompted the exploration of nanofertilizers as promising alternatives for enhancing crop production. In recent decades, nanotechnology has emerged as a highly promising and progressive field, with numerous applications in applied science and technology [3]; nanoparticles (NPs) possess unique characteristics owing to their high surface reactivity and large surface area relative to volume [3].

After dispersing as crawlers (first-instar nymphs) from maternal scales and settling on nearby leaves, female arrowhead scale insects (*Unaspis yanonensis*) become sessile and remain in this location for the remainder of their life, including development and reproduction. The primary host plant for *U. yanonensis* is the Satsuma mandarin orange, *Citrus unshiu*, which is cultivated extensively in the southwestern part of Japan as well as in China, USA, Spain, Turkey, Croatia, South Korea, and Peru [4]. The arrowhead scale typically goes through two to three generations each year in Japan [5].

Selenium (Se) has been identified as an essential element for living organisms, necessitating its inclusion in a range of diets [6]. While plants do not have a specific requirement for selenium, they derive benefits from it through enhanced antioxidant activity. At low tissue concentrations, selenium promotes plant growth, productivity, and resistance against certain abiotic stresses [7]. Recent studies on insects reveal that selenium, being chemically similar to sulfur (S), displaces sulfur, inhibits cellular metabolism, alters protein structure, and becomes toxic at high concentrations [7].

Among nanomaterials, silicon dioxide (silica) nanoparticles (SiO_2_NPs) have received significant attention for their potential applications in agriculture. While silica (SiO_2_) is considered a non-essential element for plants, it plays a crucial role in providing protection against herbivores; benefits include enhanced morphological, biochemical, and molecular defenses, thereby reducing damage to plant tissues [8,9]. In particular, the incorporation of silicon into the cell walls of leaves enhances the mechanical barrier, thereby impeding insect damage [10]. Mechanical defenses by silica-added plants can cause abrasion of the mouth parts of chewing herbivorous insects [11,12,13]. However, it is not well understood whether this applies to piercing-sucking insects like scales and to plants that do not accumulate silica (in contrast to silica-accumulating plants like rice and grasses) (but see [11,14,15]). Recently, however, it has been found that silica can also reduce feeding damage on plants that do not accumulate silica (e.g., soybean [16,17]). SiO_2_NPs can bind to the insect cuticle and subsequently to physisorb waxes and lipids, a process that ultimately leads to insect dehydration [18]. Additionally, Si enrichment in plants serves as a biochemical defense mechanism against herbivores via jasmonate-mediated inducible defenses [19].

Selenium nanoparticles (SeNPs) exhibit lower cytotoxicity than Se towards higher organisms, including humans, animals, and crops. Despite their minimal impact on these organisms, SeNPs demonstrate significant bioactivity, effectively inhibiting bacteria, fungi, and even cancer cells [20]. In agriculture, SeNPs are used as antimicrobials, nematicides, and insecticides depending on the concentration and formulation [7,21,22]. SeNPs exert toxic effects on insects due to the slow release of Se. Selenium may accumulate in an insect’s organs (the Malpighian tubules or midgut), which negatively affects the insect’s development and survival [23,24]. Recent experimental data have shown that SeNPs can have an insecticidal effect on chewers like moth larvae [25].

There have been no tests of the SiO_2_NP and SeNP effects on sessile suckers such as scale insects. Therefore, we aim to test the following hypotheses regarding the potential effects of nanoparticles (SiO_2_NPs and SeNPs) and a bulk-size material (SiO_2_) on a scale insect: SiO_2_, SiO_2_NPs, and SeNPs applied to the leaves of the Satsuma mandarin orange, *C. unshiu* (1) affect the choice behavior of a piercing-sucking insect—the arrowhead scale, *U. yanonensis*—and (2) increase leaf toughness, reducing the arrowhead scale’s density and body size. We also investigate the leaf toughness and the foliar chemical contents (SiO_2_, SeO_2_, C, and Ca) of the mandarin orange. This study is expected to shed light on the multifaceted impacts of these elements on the fruit tree and its sucking insect pest, the arrowhead scale.

## 2. Results

### 2.1. Choice Experiment with the Arrowhead Scales

**Figure 1:**
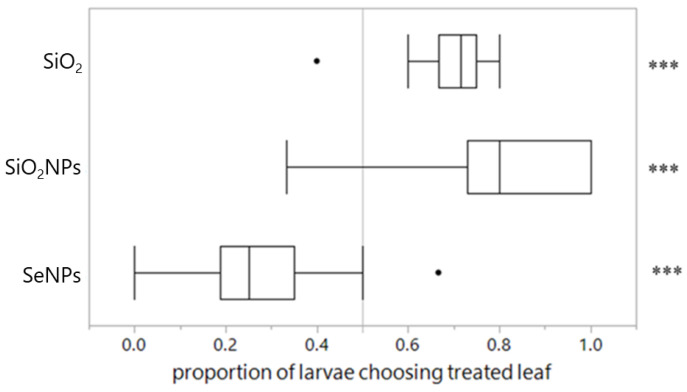
Proportion of arrowhead scales choosing a treated leaf over a paired control leaf treated with water. Paired *t*-test results of the number of scales on each leaf of the leaf pairs are shown on the right. ***: *p* < 0.001.

Arrowhead scales exhibited a strong preference for SiO_2_- and SiO_2_NP-treated leaves, while actively avoiding SeNP-treated leaves, compared with their respective paired controls (*p* < 0.001, < 0.001 and < 0.001; Table 1, Figure 1).

### 2.2. Body Size of the Arrowhead Scale

**Figure 2:**
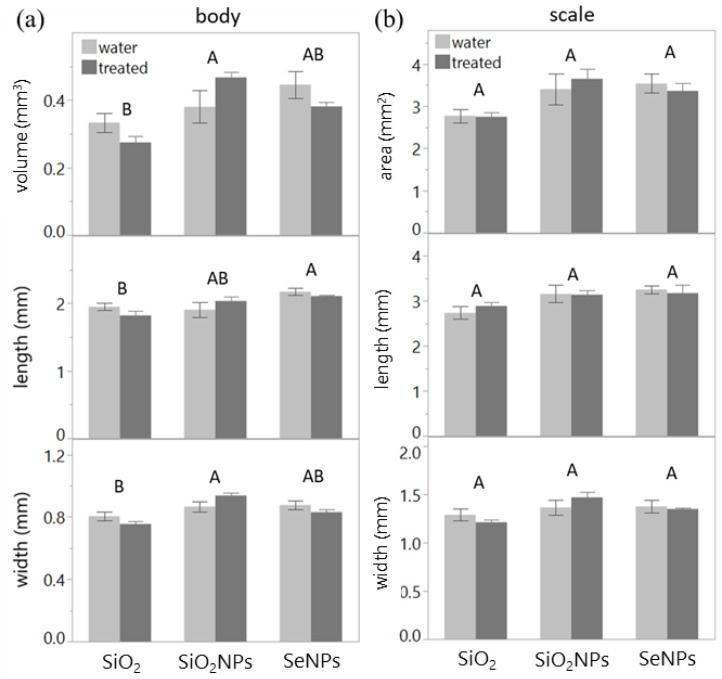
The effects of different treatments (SiO_2_, SiO_2_NPs, or SeNPs) on the size of the female arrowhead scales (mean ± SE). Shared letters above the bars indicate no significant differences. (**a**) Body volume (estimated), length, and width. (**b**) Scale area (estimated), length, and width.

The body size of female arrowhead scales was significantly reduced under the SiO_2_ treatment (volume: *p* = 0.026, length: *p* = 0.048, width: *p* = 0.009; Table 2, Figure 2a), indicating a negative effect on the development of the insects. By contrast, there were no discernible differences in scale size across all treatments (Table 2, Figure 2b).

### 2.3. Leaf Toughness

**Figure 3:**
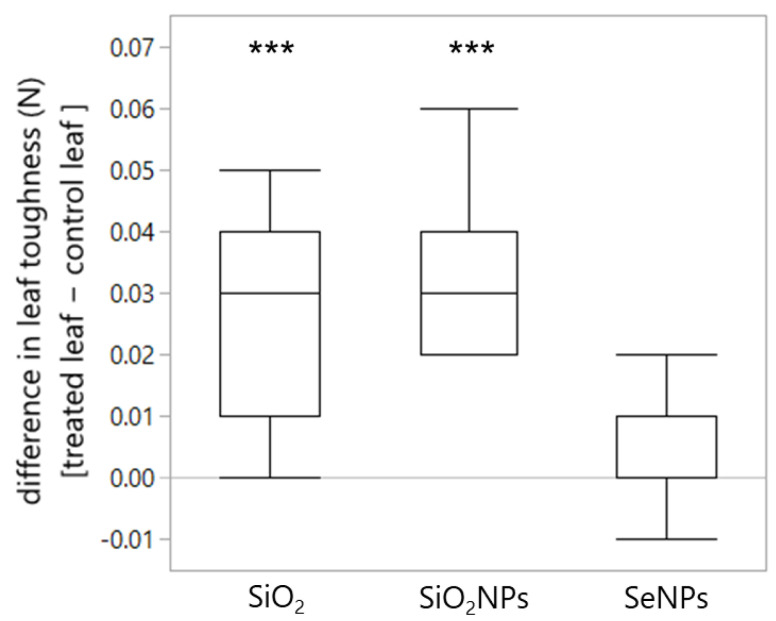
Difference in toughness between the treated leaf and the control leaf in the SiO_2_, SiO_2_NP, and SeNP treatments. ***: *p* < 0.001 in paired *t*-tests.

Both SiO_2_ and SiO_2_NPs increased the toughness of the leaf, compared with the paired water-treated leaf (*p* < 0.001, *p* < 0.001, Table 3, Figure 3). By contrast, SeNPs did not affect the toughness of the leaf (*p* = 0.221, Figure 3).

### 2.4. Leaf Chemical Contents

**Figure 4:**
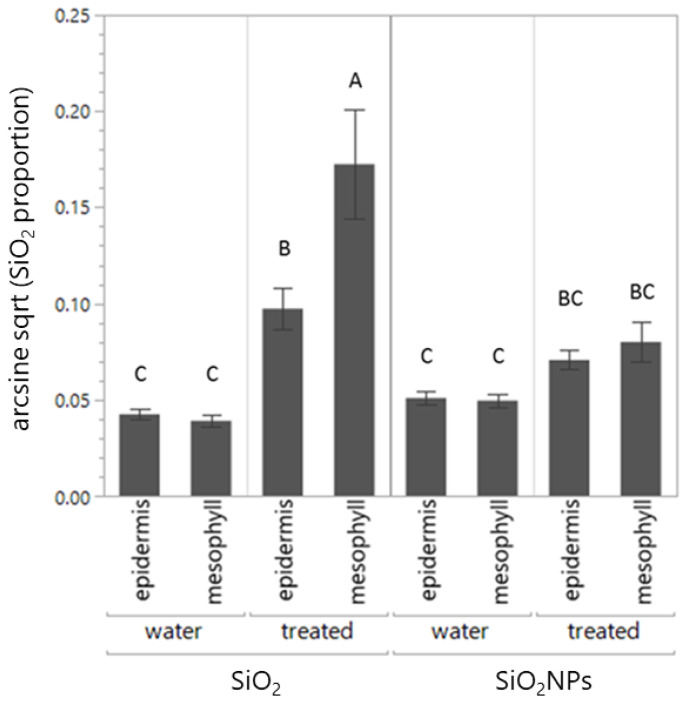
SiO_2_ content (mass %) in different leaf tissues (mean ± SE) of leaf pairs, where one leaf of the pairs was treated with SiO_2_ or SiO_2_NPs and the other leaf was treated with water. Shared letters above the bars indicate no significant differences.

In the case of SiO_2_ and SiO_2_NP treatments, there was a three-way interaction among the leaf tissue, treatment, and “control or treated leaf” factors (*p* = 0.016, Table 4, Figure 4): SiO_2_ content was higher in the mesophyll than in the epidermis (both adaxial and abaxial) in the SiO_2_ treatment (*p* < 0.001, Table 4) and in the treated leaves (*p* < 0.001, Table 4, Figure 4). When only water-treated leaves were compared, the levels of SiO_2_ content were different between the SiO_2_ and the SiO_2_NP treatments (F = 12.65, df1 = 1, df2 = 418, *p* < 0.001, Figure 4).

**Figure 5:**
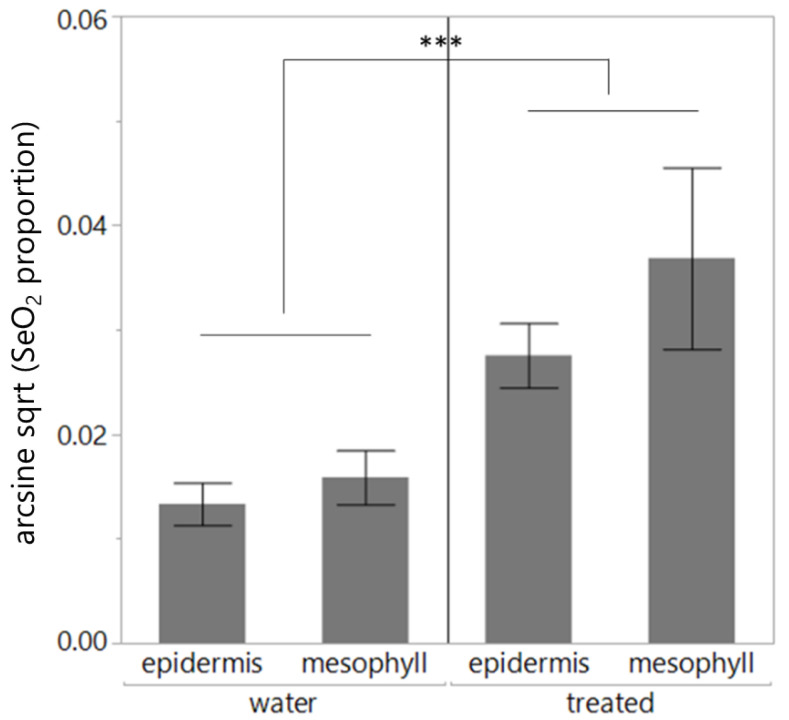
SeO_2_ content (mass %, mean ± SE) of leaf pairs, where one leaf of the pairs was treated with SeNPs and the other leaf was treated with water. ***: *p* < 0.001.

In the case of SeNP treatment, SeO_2_ content was higher in treated leaves than in control leaves (*p* < 0.001, Table 5, Figure 5), with no difference observed between the epidermis and mesophyll.

**Figure 6:**
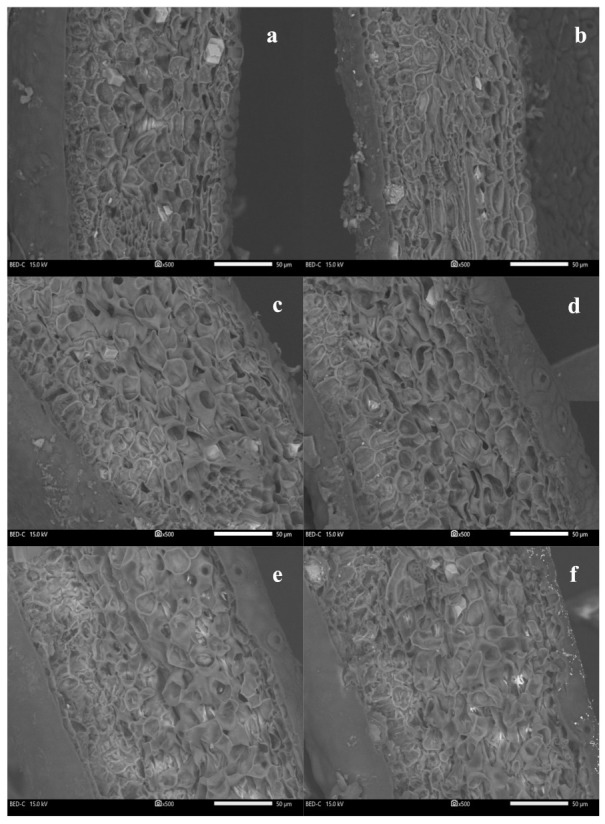
Cross-sectional SEM images of citrus leaves at a magnification of 500×. (**a**,**c**,**e**) Water-treated leaves of the same leaf pair of (**b**,**d**,**f**). (**b**) SiO_2_-treated leaf, (**d**) SiO_2_NP-treated leaf, and (**f**) SeNP-treated leaf. The scale bar = 50 μm. Leaf toughness: (**a**) 0.08 N, (**b**) 0.12 N, (**c**) 0.13 N, (**d**) 0.15 N, (**e**) 0.10 N, and (**f**) 0.11 N.

The cross-sectional images of leaves treated with SiO_2_ and SiO_2_NPs showed more densely and uniformly arranged mesophyll structure (Figure 6b,d) compared with water-treated leaves (Figure 6a,c). Conversely, there was no apparent difference in the leaf tissue structure between water-treated leaves and SeNP-treated leaves (Figure 6e,f).

### 2.5. Correlation among Scale Insect Traits and Leaf Traits

**Figure 7:**
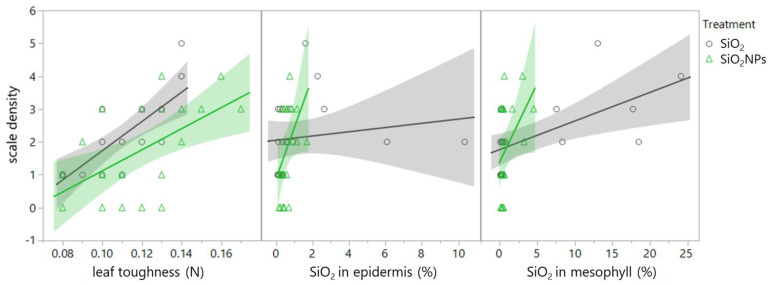
Correlation of scale density with leaf toughness (SiO_2_ treatment: *p* = 0.001, SiO_2_NP treatment: *p* = 0.004), SiO_2_ content in the epidermis (SiO_2_ treatment: *p* = 0.034, SiO_2_NP treatment: *p* = 0.006), and SiO_2_ content in the mesophyll (SiO_2_ treatment: *p* = 0.009, SiO_2_NP treatment: *p* = 0.047.

Table 6 and Table 7 present multivariate Spearman’s correlations (ρ) between the arrowhead scale variables (density and body size) and leaf properties for each treatment. Focusing on the correlations between insect and plant traits with *p* < 0.01, scale density was positively correlated with leaf toughness in both SiO_2_ and SiO_2_NP treatments (Table 6, Figure 7, ρ = 0.665 and 0.584; *p* = 0.001 and 0.004).

**Figure 8:**
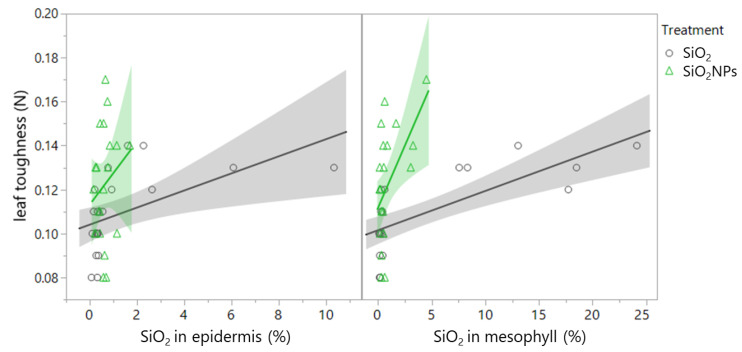
Correlation of leaf toughness with SiO_2_ content in the epidermis (SiO_2_ treatment: *p* < 0.001, SiO_2_NP treatment: *p* = 0.220) and in the mesophyll (SiO_2_ treatment: *p* < 0.001., SiO_2_NP treatment: *p* = 0.020).

**Figure A1:**
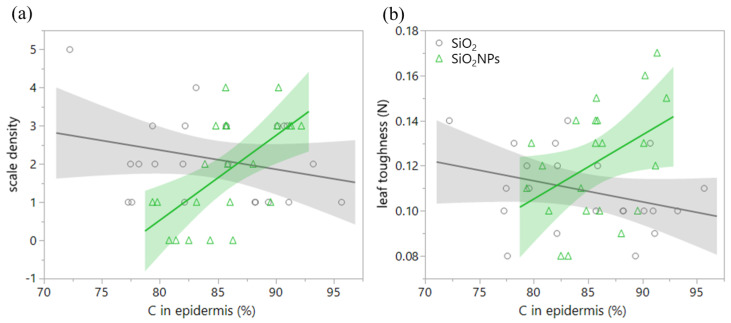
Correlations (**a**) between scale density and epidermal C content in the SiO_2_ (*p* = 0.575) and SiO_2_NP (*p* = 0.005) treatments and (**b**) between leaf toughness and epidermal C content in the SiO_2_ (*p* = 0.192) and SiO_2_NP (*p* = 0.049) treatments.

In the SiO_2_ treatment, leaf toughness was positively correlated with SiO_2_ in the epidermis (ρ = 0.687; *p* < 0.001) and SiO_2_ in the mesophyll (ρ = 0.778; *p* < 0.001) (Table 6, Figure 8). By contrast, in the SiO_2_NP treatment, leaf toughness was uncorrelated or not strongly correlated with SiO_2_ content (Table 6, epidermis: *p* = 0.020, mesophyll: *p* = 0.220). Alternatively, there were positive correlations between scale density and epidermis C content (ρ = 0.572; *p* = 0.005), as well as between epidermis C content and toughness (ρ = 0.425; *p* = 0.049) (Table A1, Figure A1).

Overall, in both silica treatments, increases in leaf SiO_2_ content were associated with an increase in toughness and an increase in arrowhead scale density.

In the SeNP treatment, no significant correlations were found between insect and plant traits (Table 7).

**Figure A2:**
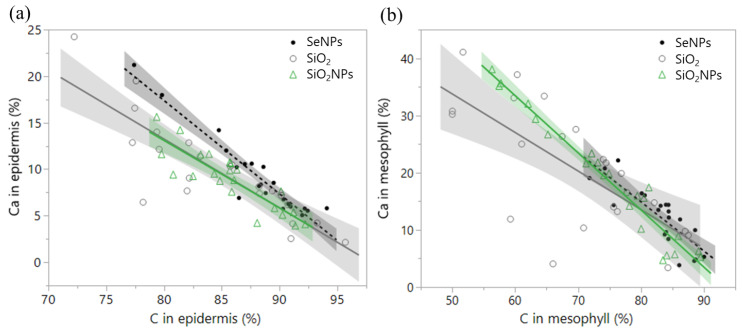
(**a**) Correlation between epidermal Ca and C content in all treatments. (**b**) Correlation between mesophyllic Ca and C content in all treatments.

Additionally, strongly negative correlations were consistently found between C and Ca content in both leaf tissues in all treatments (Table A1, Figure A2a, epidermis, ρ = −0.798, −0.826, and −0.769; *p* < 0.001, < 0.001, and < 0.001 for treatments with SiO_2_, SiO_2_NPs, and SeNPs, respectively) (Table A1, Figure A2b, mesophyll, ρ = −0.685, −0.950, and −0.795; *p* < 0.001, < 0.001, and < 0.001 for treatments with SiO_2_, SiO_2_NPs, and SeNPs, respectively).

## 3. Discussion

We tested the hypotheses that SiO_2_ and Se applied to *C. unshiu* will (1) affect the choice behavior of the arrowhead scale, *U. yanonensis*, and (2) increase leaf toughness, affecting scale density and body size. Scale insect nymphs were attracted to leaves treated with SiO_2_ and SiO_2_NPs but avoided leaves treated with SeNPs. SiO_2_ content did not differ between control (water-treated leaves) and SiO_2_NP-treated leaves but was higher in bulk-size SiO2-treated leaves compared with water-treated leaves. SiO_2_ and SiO_2_NPs increased the toughness of leaves, while SeNPs did not affect the toughness. There were positive correlations between leaf toughness and mesophyll SiO_2_ content as well as between leaf toughness and insect density per leaf in both silica treatments. In the epidermis of leaves treated with SiO_2_NPs, increased C content—rather than SiO_2_ content—was associated with increased leaf toughness.

### 3.1. Bulk SiO_2_ and SiO_2_NPs

Irrespective of particle size, SiO_2_ plays a crucial role in enhancing the toughness of plant tissues [26,27,28,29]. This is also consistent with the results obtained in this study. Intriguingly, the application of lower concentrations of nano-silica was more efficient in influencing plants compared with its bulk counterpart. This result has also been demonstrated in cucumber, for which SiO_2_NP treatments increase hardness compared with an equivalent concentration (250 mg/L) of potassium silicate (K_2_SiO_3_) [30]. These findings extend to citrus plants, highlighting the importance of nano-silica in plant physiology.

While arrowhead scales exhibited a distinct preference for leaves treated with SiO_2_ and SiO_2_NPs, the precise mechanism behind this attraction is unknown. It is plausible that the scales are drawn to treated leaves based on the emitted odors—a theory supported by earlier studies [17,27]. Alternatively, the increased leaf toughness resulting from increased SiO_2_ content may be a determining factor in the preference of arrowhead scale nymphs. This aligns with the observed feeding and oviposition preferences of other sucking hemipteran insects, such as whiteflies, which prefer thick leaves with compact vascular bundles [31]. Since thick leaves with compact vascular bundles make them tougher [32], the density of whiteflies may increase with leaf toughness. The positive correlation between scale insect density and leaf toughness challenges conventional expectations of plant resistance against insect herbivores. While the expected negative relationship holds true for chewing insects, this positive correlation is a general trend in sucking insects with piercing-sucking mouth parts like whiteflies [32]. For sessile sucking insects such as the arrowhead scale, this preference might suggest that tougher leaves provide a more secure anchoring site, thereby supporting insect survival. Although there was a strong positive correlation between leaf toughness and insect density, we could not determine whether it was tougher leaves that attracted the arrowhead scales or if the increased toughness was a result of insect feeding.

In the SiO_2_ treatment but not in the SiO_2_NP treatment, insect body volume was reduced. Similarly, reduction (although non-significant) in dry body mass and body surface area was found in the sucking insect—the rice stalk stink bug, *Tibraca limativentris*—feeding on the rice treated with 1% potassium silicate solution (20 mL per pot), which seems to be due to a higher Si content in the rice [33]. In our case, however, there was no correlation between leaf tissue Si content and scale body size, indicating that Si content in the plant is not responsible for the body size reduction. We hypothesize that more SiO_2_ particles attached to the body surface as nymphs walked on the leaves treated with the high concentration of bulk SiO_2_, leading to a smaller size through physical dehydration.

Intraspecific competition is also not a causal factor of reduced body size because there was no negative correlation between scale density and body volume (Table 6).

We observed a smaller difference in silica content between nano-treated and water-treated leaves compared with the difference between bulk-size silica and water-treated leaves. This discrepancy may be attributed to the smaller particle size of nano-silica, potentially enhancing its mobility within the plant [34]. The active or passive translocation mechanism responsible for this phenomenon remains a subject for future exploration.

### 3.2. SeNPs

This study demonstrated a strong repellent effect of Se or Se-treated plant against the arrowhead scale. This is consistent with the results of previous studies on other insects (e.g., the beet armyworm, *Spodoptera exigua*; the cabbage looper, *Trichoplusia ni*; the cabbage white, *Pieris rapae*; and the house cricket, *Acheta domestica*) [7].

Several studies have substantiated that Se can have repellent and toxic effects on various phytophagous insects and that, at the same time, both organic and inorganic Se compounds can exert toxic effects on insects to varying degrees [35]. Previous studies consistently demonstrate that Se serves as an effective repellent for herbivorous insects, negatively impacting the feeding behavior of specific species [36,37,38]. Crickets prefer to feed on leaves with low Se content [39]. Similarly, a choice experiment showed that *P. rapae* larvae strongly preferred Se-absent leaves, exhibiting higher feeding rates compared with those of Se-present leaves [40]. Laboratory studies showed that an Se-enriched diet acts as antifeedant for *S. exigua* larvae and influences their choice of plants and feeding site [41,42]. At the same time, however, Se exerts a more pronounced negative impact on the natural enemies of herbivorous insects than on the herbivores themselves, which could be attributed to a less protected body morphology [43] or the biological transfer of Se from their herbivorous hosts [44]. In recent studies, the application of specific concentrations of Se significantly influenced plant growth (*Citrus reticulata* at 150 mg/L [45]) and mitigated insect pest damage to a plant (*Atractylodes macrocephala* (Asteraceae) [46]). However, low concentrations of SeNPs (10–500 mg/L) can increase survival of pest insects (e.g., the azuki bean beetle, *Callosobruchus chinensis*, and the cowpea beetle, *C. maculatus*) [43,47,48], whereas higher concentrations of SeNPs or Se can inhibit the development and/or survival of both pest insects and their natural enemies (at 500–1000 mg/L, *C. chinensis*, *C. maculatus*, and the parasitoid—*Anisopteromalus calandrae*) [43,47,48] as well as plants such as *A. macrocephala* [46] and *Citrus reticulata* (at 200 mg/L) [45]. Therefore, the application of Se within a reasonable stoichiometric range emerges as a crucial consideration for future research.

For example, smaller doses of SeNPs might be effectively used instead of selenium, resulting in a more positive influence on agricultural crops, attributed to the presumed biosafety and bioactivity of SeNPs [49].

### 3.3. Calcium (Ca) and Carbon (C)

Both scale density and leaf toughness increased with increasing epidermal C content in the SiO_2_NP treatment, whereas leaf toughness did not increase with increasing epidermal SiO_2_. There are experimental results that show that C or both Ca and C enhance leaf toughness [50,51,52,53]. There is a negative correlation between the concentrations of Si and C in the aboveground tissues of grasses [50]. Si enhances the accumulation of C in grasses [54]. Si alone has been shown to be accumulated in the epidermis of the adaxial side of the citrus leaf, as a form of Si granules [55]. Our SEM observation indicates a morphological change in the adaxial side of the mesophyll structure. In our study, Si and C content were independent of each other in the SiO_2_NP treatment. On the contrary, in this study, there was a significant negative correlation between mesophyllic C and epidermal SiO_2_ in the SiO_2_ treatment and a marginally significant negative correlation between mesophyllic Ca and SiO_2_ in the SiO_2_NP treatment (Table A1). This might be partly due to a “dilution effect” in which an increase in C or Ca inevitably leads to a relative decline in SiO_2_ [56]. Therefore, Si and C may contribute in different ways (functional vs. structural) to increasing leaf structural toughness in *C. unshiu*. The relationship between Si and C needs further investigation.

In addition, the C and Ca content in leaves were negatively correlated in both the epidermis and mesophyll (Figure A2a,b). This is consistent with the findings in other woody plants [51]. A wide range of insects tend to reject various forms of calcium (Ca) compounds present in crops, but insects with piercing-sucking mouthparts are less affected [57,58,59]. Our present result is in line with this general trend.

This study marks a pioneering effort in comparing the impact of different particle sizes of silica on both a host plant and a pest insect. This is the first to show that silica-treated plants attract not only predators or parasitoids but also herbivores. Silica-treated plants might be used as a lure to trap scale nymphs.

## 4. Materials and Methods

### 4.1. Plant and Insect

The Satsuma mandarin orange, *Citrus unshiu* (Rutaceae), used in this study was the early ripening variety, Miyagawa-wase, which was cultivated and grown in a greenhouse. The environmental conditions were controlled at 25 ± 1 °C, 70% r.h., 450 ppm of carbon dioxide, and under natural sunlight. The potted soil was watered three times per week.

Twelve trees were planted in pots (volume: 12.8 L). The pots were filled with soil consisting of rice husk compost, coconut fiber, charcoal balls, perlite, effective microorganisms, and other components, with a pH range of 6.0–7.0.

To test preference by insects, a choice experiment was conducted as follows: Citrus leaves with female adults of the arrowhead scale, *Unaspis yanonensis* (Diaspididae), were collected from citrus trees in orchards located in Fukuoka Prefecture on 30 August 2022.

### 4.2. Reagents

We used bulk-size SiO_2_, SiO_2_NPs, and SeNPs, as well as distilled water as a control group. Each of the solutions was sonicated. The morphology of these particles was examined using a scanning electron microscope (SEM) (JSM-IT700HR, JEOL Ltd., Tokyo, Japan), operating at an accelerating voltage of 15 kV, and a transmission electron microscopy (TEM) (JEM2100HC, JEOL, Tokyo, Japan), operating at an accelerating voltage of 200 kV.

#### 4.2.1. SiO_2_ and SiO_2_NPs

The bulk-size SiO_2_ (porous silica gel; Sieweves Co., Ltd., Aichi, Japan) was prepared at 0.16 mol/L (9.61 g/L) with distilled water. This preparation forms silicic acid Si(OH)_4_, which is water soluble upon contact with water. The SiO_2_ used in the experiment had a particle size of 32.8 ± 8.7 μm (mean ± SE, n = 25, range: 3–93 μm), which was estimated from a SEM image.

SiO_2_NPs (US Research Nanomaterials, Inc., Houston, TX, USA) were prepared at 0.0016 mol/L (96.1 mg/L), which is ^1^/100th of the concentration of the bulk SiO_2_. The SiO_2_NPs used in the experiment had a particle size of 13.0 ± 0.8 nm (mean ± SE, n = 15, range: 10–19 nm), which was estimated from a TEM image.

#### 4.2.2. SeNPs

SeNPs were synthesized at room temperature through the reduction of sodium selenite (Na_2_SeO_3_) with ascorbic acid (C_6_H_8_O_6_), utilizing polysorbate 20 as a stabilizing agent [59]. The SeNPs were stored at 4 °C and used within two months of synthesis. The SeNPs had a particle size of 48.3 ± 5.5 nm (mean ± SE, n = 13, range: 23–95 nm), which was estimated from a TEM image. The concentration of the SeNPs was adjusted to 0.0016 mol/L (126 mg/L), which is consistent with the concentration of the SiO_2_NPs.

### 4.3. Experiments Using Bifoliate Leaves

To control factors such as the morphology, physiology, and genetics of the leaves in our experiments, we used bifoliate new leaves (grown in 2022) for pairwise comparisons between water-treated control leaves and chemically treated leaves. SiO_2_, SiO_2_NPs, or SeNPs were applied as follows: We chose to use new leaves (current-year leaves) located in the upper canopy to ensure an even exposure of treated leaves to sunlight. We sprayed both adaxial and abaxial surfaces of one of the bifoliate leaves once with one of the solutions (0.74 ± 0.04 mL, mean ± SD, n = 5), totaling approximately 1.48 mL per leaf. The other leaves were sprayed likewise with distilled water. The treatment was conducted only once at the beginning of the experiment. Four bifoliates (i.e., eight leaves) per tree and three trees per treatment were used; hence, each treatment–control combination was replicated 12 times.

#### 4.3.1. Choice Experiment with the Arrowhead Scales

On 30 August 2022, after the leaves has been sprayed, one leaf infested with one female adult scale collected from the orchard was placed at the point where the two leaves of a bifoliate branched, to allow the first-instar nymphs to choose between the two leaves. Forty-one days after the first appearance of the first-instar nymphs, the total number of arrowhead scales was recorded, followed by toughness measurements and EDX analyses.

#### 4.3.2. Body Size of the Arrowhead Scale

We collected arrowhead scales from the choice experiments and calculated the body volume of adult females as well as the surface area of the scales to determine the effects of the different materials on insect development. We measured the length and width of bodies and scales to the precision of 0.001 mm with a microscope (VH-5500, Keyence, Osaka, Japan) for this purpose. Given that the bodies and scales of the arrowhead scales are approximately oval, we used Yanagi and Tuda’s [60] formula for calculating volume: *V* = *πLW*^2^/12, which is half of an ellipsoid, where *L* is the main axis (i.e., length) and *W* is the minor axis (width) of the body or scale. The area of the scale was estimated using the formula *S* = *πLW*/4.

#### 4.3.3. Leaf Toughness

The toughness (in Newtons, N) of 14 leaves from each treatment was measured using a rheometer (Compac-100, Sun Scientific Co., Tokyo, Japan) at a stress rate of 60 mm/min, at three different points. The mean toughness of the three points for each leaf was used in later statistical analysis. Measurement of leaf toughness was conducted 104 days after spraying.

#### 4.3.4. Leaf Chemical Content

We obtained cross sections of leaves from the choice experiments using a razor blade, which was cleaned with ethanol before and after each use. Samples were fixed on an aluminum SEM mount covered with conductive carbon adhesive tape. The elemental composition of the samples was analyzed using a scanning electron microscope (SEM) (JSM-IT700HR) with an energy-dispersive X-ray spectrometer (EDX) (JED-2300 Analysis Station Plus, JEOL, Tokyo, Japan) at a low vacuum (30 Pa), 15 kV accelerating voltage, and 500× magnification. We measured three points within the epidermis of both the adaxial and abaxial surfaces and four points within the mesophyll. The SEM–EDX analysis was conducted on the same day as the toughness measurements.

### 4.4. Statistical Analyses

The number of arrowhead scales and the leaf toughness between bifoliate leaves were compared using paired *t*-tests for each treatment. The SiO_2_ or SeO_2_ content (mass %, mean per tissue per leaf) in leaves treated with SiO_2_, SiO_2_NPs, and SeNPs were arcsine square-root transformed and then analyzed using a general linear model; treatment (only for the two silica), leaf tissue, control or treated leaf, tree ID (nested within treatment), and leaf pair ID (nested within tree ID and treatment) were used as explanatory variables. Furthermore, SiO_2_ content in water-treated leaves with their paired leaves treated with SiO_2_ or SiO_2_NPs were compared between SiO_2_ and SiO_2_NP treatments, using a subset of the general linear model. Multivariate correlations among scale density, body volume and scale area (both mean per leaf), leaf toughness (mean per leaf), and the content (mass %, mean per tissue per leaf) of treated elements (SiO_2_ or SeO_2_), C, and Ca in leaf epidermis and mesophyll were tested using nonparametric Spearman correlations. All statistical analyses were performed using JMP, version 13.0.

## Figures and Tables

**Table 1:** Paired *t*-test results on the number of arrowhead scales that chose either the control or the treated bifoliate leaf when one of the bifoliate leaves was treated with SiO_2_, SiO_2_NPs, or SeNPs and the other treated with water.

Treatment	df	*t*	*p*
SiO_2_	12	5.28	<0.001
SiO_2_NPs	13	6.75	<0.001
SeNPs	13	−5.26	<0.001

**Table 2:** General linear model results of different treatments (SiO_2_, SiO_2_NPs, or SeNPs) on female body volume and scale area of the arrowhead scale, *Unaspis yanonensis*.

		Source	df1	df2	*F*	*p*
Body	Length	Treatment	2	5	5.90	0.048
		Water or treated [tree, treatment, leaf]	7	5	0.48	0.818
		Tree [treatment]	6	5	1.57	0.318
		Leaf [treatment, tree]	15	5	1.00	0.550
	Width	Treatment	2	5	13.38	0.009
		Water or treated [tree, treatment, leaf]	7	5	1.01	0.505
		Tree [treatment]	6	5	1.77	0.275
		Leaf [treatment, tree]	15	5	1.43	0.368
	Volume	Treatment	2	5	8.30	0.026
		Water or treated [tree, treatment, leaf]	7	5	0.59	0.733
		Tree [treatment]	6	5	1.85	0.259
		Leaf [treatment, tree]	15	5	1.09	0.505
Scale	Length	Treatment	2	5	5.53	0.054
		Water or treated [tree, treatment, leaf]	7	5	0.67	0.685
		Tree [treatment]	6	5	2.51	0.165
		Leaf [treatment, tree]	15	5	1.18	0.461
	Width	Treatment	2	5	2.51	0.176
		Water or treated [tree, treatment, leaf]	7	5	0.61	0.718
		Tree [treatment]	6	5	0.70	0.663
		Leaf [treatment, tree]	15	5	0.75	0.693
	Area	Treatment	2	5	2.99	0.140
		Water or treated [tree, treatment, leaf]	7	5	0.28	0.924
		Tree [treatment]	6	5	0.96	0.530
		Leaf [treatment, tree]	15	5	0.64	0.769

**Table 3:** Paired *t*-tests comparing the leaf toughness of paired leaves that were sprayed with either water or a chemical solution.

Treatment	df	*t*	*p*
SiO_2_	10	5.60	<0.001
SiO_2_NPs	10	8.86	<0.001
SeNPs	10	1.30	0.221

**Table 4:** General linear model analysis on the SiO_2_ concentration (mass %) in different leaf tissues (epidermis or mesophyll) after the foliar spray of SiO_2_ or SiO_2_NPs. Tree ID was nested within treatment, and leaf ID was nested within tree ID and treatment.

Source	df1	df2	*F*	*p*
Treatment	1	852	15.74	<0.001
Leaf tissue	1	852	7.99	0.005
Control or treated leaf	1	852	71.58	<0.001
Leaf tissue × Treatment	1	852	5.11	0.024
Leaf tissue × Control or treated leaf	1	852	24.04	<0.001
Control or treated leaf × Treatment	1	852	10.04	0.001
Leaf tissue × Treatment × Control or treated leaf	1	852	5.82	0.016
Tree ID [treatment]	4	852	13.19	<0.001
Leaf pair ID [tree ID, treatment]	16	852	2.67	0.001

**Table 5:** General linear model analysis on the SeO_2_ content (mass %) in different leaf tissues (epidermis or mesophyll) after foliar spray of SeNPs. Leaf ID was nested within tree ID.

Source	df1	df2	*F*	*p*
Leaf tissue	1	426	1.93	0.165
Control or treated leaf	1	426	17.07	<0.001
Leaf tissue × Control or treated leaf	1	426	0.63	0.429
Tree ID	2	426	11.11	<0.001
Leaf pair ID [tree ID]	8	426	0.55	0.816

**Table 6:** Multivariate correlation analysis on the leafwise parameters: SiO_2_ content (mass %) in different leaf tissues (epidermis or mesophyll); scale body volume and density; and leaf toughness after the foliar spray of SiO_2_ or SiO_2_NPs. Italic: 0.01 < *p* < 0.05, bold & italic: 0.001 < *p* < 0.01, and bold: *p* < 0.001. The number of pairs of samples is shown in parentheses.

		Treatment			
		SiO_2_		SiO_2_NPs	
Variable 1	Variable 2	Spearman ρ	*p*	Spearman ρ	*p*
body volume	scale density	0.285	0.425 (10)	0.657	0.109 (7)
scale area	scale density	0.374	0.287 (10)	0.558	0.193 (7)
scale area	body volume	*0.733*	0.016 (10)	0.679	0.094 (7)
SiO_2_ in epidermis	scale density	*0.454*	0.034 (22)	***0.565***	0.006 (22)
SiO_2_ in epidermis	body volume	0.127	0.726 (10)	0.286	0.535 (7)
SiO_2_ in epidermis	scale area	0.212	0.556 (10)	−0.036	0.939 (7)
SiO_2_ in mesophyll	scale density	***0.543***	0.009 (22)	*0.428*	0.047 (22)
SiO_2_ in mesophyll	body volume	0.394	0.260 (10)	0.500	0.253 (7)
SiO_2_ in mesophyll	scale area	0.467	0.174 (10)	0.071	0.879 (7)
SiO_2_ in mesophyll	SiO_2_ in epidermis	**0.755**	<0.001 (22)	*0.529*	0.011 (22)
leaf toughness	scale density	***0.665***	0.001 (22)	***0.584***	0.004 (22)
leaf toughness	body volume	0.340	0.337 (10)	0.360	0.427 (7)
leaf toughness	scale area	0.377	0.283 (10)	0.036	0.939 (7)
leaf toughness	SiO_2_ in epidermis	**0.687**	<0.001 (22)	0.273	0.220 (22)
leaf toughness	SiO_2_ in mesophyll	**0.778**	<0.001 (22)	*0.491*	0.020 (22)

**Table 7:** Multivariate correlation analysis on the leafwise parameters: SeO_2_ content (mass %) in different leaf tissues (epidermis or mesophyll); scale body volume and density; and leaf toughness after foliar spray of SeNPs. Bold & italic: 0.001 < *p* < 0.01. The number of pairs of samples is shown in parentheses.

		SeNP Treatment	
Variable 1	Variable 2	Spearman ρ	*p*
body volume	scale density	0.267	0.562 (7)
scale area	scale density	0.535	0.216 (7)
scale area	body volume	0.643	0.119 (7)
SeO_2_ in epidermis	scale density	−0.396	0.068 (22)
SeO_2_ in epidermis	body volume	0.286	0.535 (7)
SeO_2_ in epidermis	scale area	−0.107	0.819 (7)
SeO_2_ in mesophyll	scale density	−0.202	0.367 (22)
SeO_2_ in mesophyll	body volume	−0.321	0.482 (7)
SeO_2_ in mesophyll	scale area	−0.643	0.119 (7)
SeO_2_ in mesophyll	SeO_2_ in epidermis	***0.638***	0.001 (22)
leaf toughness	scale density	0.137	0.951 (22)
leaf toughness	body volume	−0.319	0.486 (7)
leaf toughness	scale area	−0.179	0.701 (7)
leaf toughness	SeO_2_ in epidermis	0.260	0.242 (22)
leaf toughness	SeO_2_ in mesophyll	0.035	0.876 (22)

## Data Availability

Data sets are available upon reasonable request from the corresponding authors. The data are not publicly available due to our on-going analyses.

## Appendix A

**Table A1:** Multivariate correlation analysis on the leafwise parameters: Ca and C content (mass %) in different leaf tissues (epidermis or mesophyll), scale insect body volume and scale area, scale insect density, and leaf toughness after foliar spray of SiO_2_ or SiO_2_NPs. Italic: 0.01 < *p* < 0.05, bold & italic: 0.001 < *p* < 0.01, and bold: *p* < 0.001. The number of samples is shown in parentheses.

		Treatment			
		SiO_2_		SiO_2_NPs	
Variable 1	Variable 2	Spearman ρ	*p*	Spearman ρ	*p*
Ca in epidermis	scale density	0.128	0.580 (22)	−0.404	0.063 (22)
Ca in epidermis	body volume	0.309	0.385 (10)	−0.429	0.337 (7)
Ca in epidermis	scale area	0.442	0.200 (10)	−0.500	0.253 (7)
Ca in epidermis	SiO_2_ in epidermis	0.170	0.450 (22)	−0.109	0.629 (22)
Ca in epidermis	SiO_2_ in mesophyll	−0.044	0.848 (22)	−0.036	0.875 (22)
Ca in epidermis	leaf toughness	0.121	0.592 (22)	−0.202	0.367 (22)
Ca in mesophyll	scale density	−0.305	0.168 (22)	−0.305	0.168 (22)
Ca in mesophyll	body volume	0.297	0.405 (10)	−0.214	0.645 (7)
Ca in mesophyll	scale area	0.212	0.556 (10)	−0.036	0.939 (7)
Ca in mesophyll	SiO_2_ in epidermis	−0.073	0.747 (22)	−0.151	0.503 (22)
Ca in mesophyll	SiO_2_ in mesophyll	−0.180	0.423 (22)	−0.417	0.053 (22)
Ca in mesophyll	leaf toughness	−0.266	0.232 (22)	−0.404	0.062 (22)
Ca in mesophyll	Ca in epidermis	0.333	0.131 (22)	0.102	0.651 (22)
C in epidermis	scale density	−0.127	0.575 (22)	***0.572***	0.005 (22)
C in epidermis	body volume	−0.491	0.150 (10)	0.464	0.294 (7)
C in epidermis	scale area	−0.612	0.060 (10)	0.536	0.215 (7)
C in epidermis	SiO_2_ in epidermis	−0.402	0.064 (22)	0.073	0.747 (22)
C in epidermis	SiO_2_ in mesophyll	−0.162	0.471 (22)	0.058	0.797 (22)
C in epidermis	leaf toughness	−0.289	0.192 (22)	*0.425*	0.049 (22)
C in epidermis	Ca in epidermis	**−0.** **863**	<0.001 (22)	**−0.8** **30**	<0.001 (22)
C in epidermis	Ca in mesophyll	−0.334	0.129 (22)	−0.196	0.382 (22)
C in mesophyll	scale density	−0.007	0.975 (22)	0.293	0.186 (22)
C in mesophyll	body volume	−0.503	0.138 (10)	0.179	0.702 (7)
C in mesophyll	scale area	−0.285	0.425 (10)	0.071	0.879 (7)
C in mesophyll	SiO_2_ in epidermis	*−0.438*	0.042 (22)	0.114	0.615 (22)
C in mesophyll	SiO_2_ in mesophyll	−0.294	0.184 (22)	0.275	0.216 (22)
C in mesophyll	leaf toughness	−0.135	0.548 (22)	0.326	0.139 (22)
C in mesophyll	Ca in epidermis	−0.265	0.234 (22)	−0.015	0.946 (22)
C in mesophyll	Ca in mesophyll	**−0.685**	<0.001 (22)	**−0.950**	<0.001 (22)
C in mesophyll	C in epidermis	0.378	0.083 (22)	0.165	0.462 (22)

**Table A2:** Multivariate correlation analysis on the leafwise parameters: Ca and C content (mass %) in different leaf tissues (epidermis or mesophyll), scale insect body volume and scale area, insect density, and leaf toughness after foliar spray of SeNPs. Italic: 0.01 < *p* < 0.05, and bold: *p* < 0.001. The number of samples is shown in parentheses.

		SeNP Treatment	
Variable 1	Variable 2	Spearman ρ	*p*
Ca in epidermis	scale density	−0.285	0.198 (22)
Ca in epidermis	body volume	−0.536	0.215 (7)
Ca in epidermis	scale area	−0.750	0.052 (7)
Ca in epidermis	SeO_2_ in epidermis	0.047	0.836 (22)
Ca in epidermis	SeO_2_ in mesophyll	−0.027	0.903 (22)
Ca in epidermis	leaf toughness	0.366	0.094 (22)
Ca in mesophyll	scale density	0.170	0.449 (22)
Ca in mesophyll	body volume	0.286	0.535 (7)
Ca in mesophyll	scale area	−0.250	0.589 (7)
Ca in mesophyll	SeO_2_ in epidermis	0.012	0.958 (22)
Ca in mesophyll	SeO_2_ in mesophyll	−0.197	0.379 (22)
Ca in mesophyll	leaf toughness	−0.153	0.498 (22)
Ca in mesophyll	Ca in epidermis	0.231	0.301 (22)
C in epidermis	scale density	0.140	0.535 (22)
C in epidermis	body volume	0.500	0.253 (7)
C in epidermis	scale area	0.714	0.071 (7)
C in epidermis	SeO_2_ in epidermis	0.012	0.958 (22)
C in epidermis	SeO_2_ in mesophyll	0.185	0.409 (22)
C in epidermis	leaf toughness	−0.395	0.069 (22)
C in epidermis	Ca in epidermis	**−0.** **893**	<0.001 (22)
C in epidermis	Ca in mesophyll	−0.064	0.778 (22)
C in mesophyll	scale density	*−0.4* *94*	0.020 (22)
C in mesophyll	body volume	−0.286	0.535 (7)
C in mesophyll	scale area	0.000	1.000 (7)
C in mesophyll	SeO_2_ in epidermis	0.065	0.774 (22)
C in mesophyll	SeO_2_ in mesophyll	0.289	0.192 (22)
C in mesophyll	leaf toughness	−0.054	0.812 (22)
C in mesophyll	Ca in epidermis	−0.099	0.662 (22)
C in mesophyll	Ca in mesophyll	**−0.** **822**	<0.001 (22)
C in mesophyll	C in epidermis	0.065	0.774 (22)
